# International health professional perspectives of using the Cognitive Orientation to daily Occupational Performance approach in routine practice

**DOI:** 10.3389/fresc.2026.1704831

**Published:** 2026-02-26

**Authors:** Hortensia Gimeno, Ruth Swanton, Elspeth Froude

**Affiliations:** 1 Bone and Joint Health, Blizard Institute, Queen Mary University of London, London, United Kingdom; 2 The Royal London Hospital and Tower Hamlets Community Children’s Therapy Services, Barts Health NHS Trust, London, United Kingdom; 3 Occupational Therapy Department, Mercy University Hospital, Cork, Ireland; 4 School of Allied Health, Faculty of Health Sciences, Australian Catholic University, Sydney, NSW, Australia

**Keywords:** Cognitive Orientation to daily Occupational Performance, meta-cognition, routine practice, strategy use, survey

## Abstract

**Background:**

The Cognitive Orientation to daily Occupational Performance (CO-OP) Approach is an evidence-based intervention that utilizes a problem-solving approach to achieve client-chosen goals. Training is available to support professionals in learning and using the approach effectively. Therapists’ perspectives of using the approach in practice post training has not been reported.

**Methods:**

Perspectives of CO-OP trained health professionals were gathered using an international online survey. Closed and Likert scale questions were analyzed using descriptive statistics, and free-text responses were coded using content analysis.

**Results:**

The dataset compiled responses from 181 participants across 6 continents. CO-OP training was perceived positively, reporting it prepared them well for using CO-OP. However, 66% would like further support to use the approach. Of the recommended 10 individual sessions, only 1/3 of participants provided the recommended dosage. Despite the Canadian Occupational Performance Measure (COPM) and the Performance Quality Rating Scale (PQRS) being prescribed outcome measures in CO-OP, only 63% of respondents used the COPM and 23% the PQRS.

**Conclusion:**

Education and training are important to implement evidence-based interventions, but do not always lead to delivering the approach with fidelity. Future research should focus on how to support CO-OP implementation with fidelity, including process evaluation to understand clinical contexts and evaluate its effectiveness when delivered by clinicians.

## 1 Introduction

The Cognitive Orientation to daily Occupational Performance (CO-OP) Approach was developed in the early 1990's specifically for children with developmental coordination disorder (DCD), by Angela Mandich and Helene Polatajko, both occupational therapists from Canada. CO-OP is an evidence-based intervention that utilizes a cognitive problem-solving approach for children and adults to achieve individual goals that apply to movement problems and everyday activities important to them (1). While the approach was initially developed for use with children with DCD (2), evidence for the effectiveness of CO-OP for a range of child, youth and adult populations, such as neurodevelopmental disorders other than DCD (3), cerebral palsy (4), brain injury (5, 6), and stroke (7), is growing (8). As the evidence base grew for the effectiveness of the approach, training was provided to occupational therapists worldwide. Over time, this broadened to other health professionals who work with people with motor disorders, such as physiotherapists. A recent review of the use of the approach with children and young people found 38 original studies reporting on 486 participants with an average age range of 7–12, with most studies including children and young people with DCD (9). In adults with stroke, a recent systematic review identified four randomized controlled trials (RCTs) and three pilot RCTs with inconsistent results reported for trained and untrained goals (7).

CO-OP enables children, young people and adults to use a problem-solving strategy to discover solutions and strategies they can apply to achieve their goals. Following therapy, individuals can continue to use the problem-solving strategy to solve performance problems by themselves without needing ongoing therapy. This differs from interventions that focus on “fixing” the underlying movement issues (body structures and function), which frequently don’t lead to changes in activity and participation (10, 11). It is highly recommended that therapists who implement CO-OP in their clinical and/or research practices take a workshop led by a certified CO-OP instructor. The CO-OP approach is governed by ICAN—International Cognitive Approaches Network (ICAN), based in Canada, which certifies occupational therapists to be CO-OP instructors globally. These certified CO-OP instructors can then train interested health professionals (i.e., occupational therapists, physiotherapists, or exercise physiologists) to implement the approach according to ICAN's standards. This training provides the background and underlying theories to the approach and the key elements for implementation and coaching is provided during an activity to practice the approach.

The four objectives of the CO-OP intervention are 1. Skill acquisition, 2. Cognitive strategy use, 3. Generalization, and 4. Transfer. There are five core elements in CO-OP which include (i) client-chosen, occupation-based goals, (ii) dynamic performance analysis, (iii) cognitive strategy use, (iv) guided discovery, and (v) enabling principles. The suggested dosage, based on available evidence, for individual delivery of the CO-OP approach is between 10 and 12 sessions, and CO-OP delivered in group settings dosage can vary from 8 to 20 sessions (9). Treatment fidelity can be checked by using an available CO-OP fidelity checklist, available at https://www.icancoop.org.

In a knowledge translation study across five stroke rehabilitation units in Toronto (12), clinician experiences with implementing the approach in their clinical setting highlighted challenges and successes, including organisational support, the development of experiential evidence, and the varying perspectives of clinicians on the approach's effectiveness (13). However, to date, no studies have specifically investigated the outcomes of CO-OP training from the perspective of therapists.

An important question is what strategies are effective in supporting knowledge uptake, and these strategies need to be tailored to the targeted population and setting (14). Gaining clinicians' perspectives on using the CO-OP approach in their work settings, with a focus on practice gaps is an important strategy for identifying barriers and enablers to implementation. This international study aimed to explore how health professionals use the CO-OP approach in practice following formal training, including dosage, populations served, clinician confidence, and implications for knowledge translation and implementation. Thefollowing research questions were addressed:

- What are health professionals’ perspectives on using CO-OP in practice, and what are the perceived practice gaps?
- What are the health professional perspectives of the usefulness of CO-OP training?

## 2 Methods

### 2.1 Design

An international online cross-sectional survey design with quantitative and qualitative elements was used. Ethics approval was obtained through the Australian Catholic University Human Research Ethics Committee (2022-2478E).

### 2.2 Participants

Inclusion criteria were health care professionals who had completed a certified 2- or 3-day CO-OP training course (in-person/online) anywhere in the world. We aimed to recruit 100 participants. The sample size was guided by established survey methodology indicating that approximately 100 respondents provide acceptable precision for exploratory estimation (15). Clinician implementation surveys in rehabilitation commonly report completed samples in the range of approximately 100 respondents, including mixed occupational therapy and physiotherapy surveys examining clinicians' experiences and perceptions of intervention implementation in practice (16). Given the absence of a definitive sampling frame for CO-OP–trained clinicians internationally, the target sample size was therefore also informed by pragmatic feasibility considerations.

### 2.3 Recruitment

Purposive sampling with recruitment through ICAN, via country CO-OP instructor coordinators, and CO-OP instructors; host organisations delivering CO-OP courses via direct emails and snowball sampling within the authors' professional networks; and via social media such as Twitter/X using a survey announcement.

### 2.4 Data collection

Data were collected using an online questionnaire with Qualtrics software via an anonymous link or a QR code from October 2022 until March 2023. Consent was obtained within the survey. Participants were informed about the length of the survey (15–20 min), which data will be stored, the research team, and the purpose of the study.

Survey items were designed to capture key implementation domains relevant to post-training use of CO-OP, including adoption, fidelity, adaptation, feasibility, acceptability, and perceived barriers and facilitators. Items addressed frequency and mode of use, dosage and service configurations, clinician confidence, outcome evaluation, and factors influencing implementation. This domain-based structure reflects common themes raised by clinicians during CO-OP training and post-training discussions and is consistent with exploratory implementation research. The full survey is provided as Supplementary Material. A copy of the survey is shown in Supplementary Material. The survey format included multiple-choice and Likert-scale questions and some free-text responses to elicit data on perceived barriers and facilitators to applying the approach in routine clinical practice. Adaptive questioning (certain items only conditionally displayed based on the response to other items) was used to reduce the number and complexity of questions. The questionnaire content and usability were piloted with three CO-OP trained therapists, with subsequent minor amendments made. The online questionnaire was translated into French, Dutch, and Spanish, using the automatic Qualtrics software translation feature and checked for accuracy by native-level speakers. Translation to these languages was pragmatic and based on the languages spoken by research team.

Email and social media reminders were used to increase the response rate. No incentives were used to increase the response rate. All responses were anonymous. Qualtrics platform uses IP addresses on participants' computers to identify potential duplicate entries. Data were exported from Qualtrics to the Statistical Package for Social Sciences (SPSS) v28 for analysis. Data is reported using the Checklist for Reporting Results of Internet E-Surveys (CHERRIES) (17).

### 2.5 Data analysis

Quantitative data from closed-ended survey items were analyzed using descriptive statistics. Categorical variables (e.g., profession, country, populations served, barriers and facilitators) were summarized using counts and percentages. Ordinal Likert-scale items were summarized using measures of central tendency and dispersion (means and standard deviations). For continuous variables (e.g., number of sessions), medians and interquartile ranges were also reported. Free text responses were analyzed using content analysis (18). Free-text responses were examined to identify relevant phrases or statements related to the study aims. These were summarized and coded inductively, with codes compared and grouped into categories reflecting recurring barriers, facilitators, and contextual factors influencing CO-OP implementation. Initial coding was conducted by one of the researchers (RS), with categories and interpretations reviewed and discussed with the two additional researchers (EF, HG) to enhance analytic rigor and reduce individual basis. Quantitative and qualitative findings were integrated at the interpretation stage to provide a comprehensive description of CO-OP implementation in practice.

## 3 Results

A total of 191 questionnaires were received, of which (*n* = 156, 82%) were fully completed. Questionnaires with no data included (*n* = 3, 1%) or with less than 25% of the survey completed (*n* = 10, 5%) were removed before analysis, resulting in 181 surveys used for final analysis.

### 3.1 Demographics

The main group responding to the survey were occupational therapists (*n* = 170, 94%), six physiotherapists, 2 speech and language therapists, 2 psycho-motor therapists, and 1 special education teacher. The average years since graduating in their professions was 15.7 years (SD: 10.2) ranging from 1 to 40 years. The majority of answers were provided by allied health professionals from the United Kingdom (*n* = 64, 35%), The Netherlands (*n* = 26, 14%), and Australia (*n* = 25, 14%) but with representation from North America, Europe, and Asia.

Almost half of the respondents had completed their CO-OP training in the past 1–2 years (*n* = 83, 46%) the mean and standard deviation (SD) of years of professional practice since graduation were 15.66 (10.24), indicating most healthcare professionals had worked for some time before completing formal CO-OP training. A quarter of participants had trained in CO-OP in the past 3–5 years (*n* = 40, 22%), and a smaller number of participants had trained 6–10 years ago (*n* = 26, 14%) and the same for training received >10 years ago (*n* = 26, 14%). Only a small proportion of respondents had never had formal CO-OP training (*n* = 6, 3%). The largest proportion of respondents had used CO-OP with children (*n* = 159/181, 88%) and a smaller proportion of participants had used the approach with young people (*n* = 26, 14%), adults (*n* = 31, 17%), and older people above 65 years of age (*n* = 17, 9%).

The majority of respondents identified using CO-OP with children and young people with 86% of respondents (*n* = 136) identifying DCD as the most prevalent condition seen for CO-OP intervention, followed by autism with *n* = 103, 65%, and ADHD in *n* = 88, 55%. A full outline of number of participants applying CO-OP to different health conditions and percentages is shown in Table 1.

**Table 1:** Participants applying CO-OP to different health conditions.

Children and young people (*n* = 185) Number (%)	Adults and older people (*n* = 48) Number/total
DCD: 136 (74%)	Stroke: 27 (56%)
Autism: 103 (56%)	ABI: 18 (38%)
ADHD: 88 (48%)	Autism: 4 (8%)
CP: 61 (33%)	Schizophrenia: 4 (8%)
Any other ID: 32 (17%)	MS: 3 (6%)
ABI: 28 (15%)	Parkinsons: 3 (6%)
Other: 23 (12%)	Long Covid: 3 (6%)
Fetal Alcohol Spectrum Disorder 5 (3%)	ADHD: 3 (6%)
Spina Bifida 5 (3%)	Bipolar: 3 (6%)
	Fatigue: 2 (4%)
	Depression: 2 (4%)
	Intellectual disabilities: 3 (6%)
	Anxiety: 1
	FND: 1
	CP: 1
	Other condition: medical complex, oncology: 1

DCD, developmental coordination disorder; ADHD, attention deficit and hyperactivity disorder; CP, cerebral palsy; ID, intellectual disability; ABI, acquired brain injury; MS, multiple sclerosis; FND, functional neurological disorder. “Other” Include the following conditions: anxiety, post-traumatic stress, developmental language disorder, dystonia, global developmental delay, long covid, specific learning difficulties, neuromuscular disorders, rare disorders, and sensory processing problems.

Most respondents were employed in government-funded institutions (e.g., National Health System in the UK), *n* = 109, followed by private providers/independent practice in 47 cases. Twenty-five people responded “other” and included not-for-profit institutions or schools. The majority of participants worked with childhood populations <6 years (*n* = 120), 6–11 years (*n* = 141), and 12–16 years (*n* = 128), then youth populations, 16–19 years (*n* = 88) and for adults19–25 years (*n* = 35), 26–65years (*n* = 41) and older people >65 years (27).

### 3.2 Usefulness of CO-OP training

Participants positively perceived CO-OP training, reporting it prepared them to understand the approach and the application to their current practice (*n* = 28, 16%) extremely well, (*n* = 92, 52%) very well, (*n* = 62, 35%) moderately well and (*n* = 11, 6%) slightly well. Out of the 176 people answering this question, 140 (80%) reported they currently used the approach in their practice. Self-efficacy in delivering the approach was measured with a 0–100 scale (19) with a mean of 70, SD (21.82). Most respondents found CO-OP training prepared them very well (91/181), moderately well (44/181), extremely well (29/181) to understand the approach, and only 11/181 reported slightly well, and 1 not well at all.

Only 55% of participants (*n* = 99) had accessed information on the ICAN.org website, with the majority (71%) finding it somewhat useful (*n* = 13 extremely useful, *n* = 43 moderately useful, and *n* = 15 slightly useful). The purpose of accessing ICAN website varied and was primarily around accessing information on training (*n* = 23), seeking information resources on the approach (*n* = 48), providing information for clients, parents, and/or students (*n* = 12), and increasing knowledge on the approach (*n* = 22).

### 3.3 Translation of CO-OP into practice

Knowledge translation is the process by which research knowledge is synthesized, communicated, and applied in real-world contexts to bridge the gap between evidence and practice. For those participants not using the CO-OP approach, (*n* = 36, 20%) reported rarely having a client on their caseload with whom they were using CO-OP. The majority of respondents (*n* = 84, 60%) often or always had a client with whom they were using CO-OP, and (*n* = 48, 34%) reported occasionally having a client in their caseload to use the approach with. Reasons not to use the approach included (i) time factors to implement the approach with participants only using some components of the approach, (ii) challenges with identifying functional goals with their clients (e.g., “*I find it difficult for clients to identify functional, meaningful tasks.Most often they say, I want to improve my arm*”, or “*Some children struggle to articulate goals and often rely on parents or schools to decide what they should work on*”, (iii) lack of confidence in applying the approach, (iv) not having appropriate/suitable clients such as children with profound learning difficulties or children with autism who participants reported required explicit teaching. When asked if they would use it in the future themajority agreed they would use it if the client met the criteria and could apply the problem-solving approach. From the 139 available answers of how frequently therapists used the full CO-OP approach, 29 participants responded always having a client on their caseload using the approach (21%), 48 (35%) occasionally having a client using the approach, 55 (40%) often having a client in caseload and 7 (5%) rarely having someone in their caseload with whom using the CO-OP approach.When CO-OP was delivered, participants provided mainly individual therapy (*n* = 123, 88%), with some offering both individual and group-based intervention (*n* = 14, 10%), and a small proportion only using a group-based mode of delivery (*n* = 2, 1%). Dosage varied when providing both, individual and group modes of delivery. For individual delivery CO-OP sessions, a third of respondents provided at least 10 sessions of CO-OP (*n* = 43/130, 33%) with another third using either six sessions or less (*n* = 35/130, 32%), mean and standard deviation (SD) 8.5(3.9), median and interquartile range (IQR) 8(6–10). Some reported number of sessions provided depended on the child (*n* = 18/130, 14%), and about 27 respondents reported less than 10 sessions but more than 6 (*n* = 27/130, 21%). The dosage for CO-OP delivered in groups also varied greatly. Two participants reported to run groups for 20 sessions, one participant ran 12–16 sessions, two participants ran groups for 4 sessions, four participants ran 4–6 sessions, and two participants ran 7–8 sessions.Groups were delivered at service level differently across the year, mean and SD: 8.2(5.9), median and IQR: 6 (5–7) with four participants delivering groups once a year, four delivering 3–4 per year, one participant reporting to run six groups per year, and another reporting 5 per year. The ratio of staff trained and untrained and children in groups also varied with different models being used. Six participants responded they conducted groups with two trained CO-OP therapists for 4–6 children in each group. Three participants responded to using one trained therapist and one or two support workers for groups of 5–6 children. Goals for CO-OP when set up with participants in a group, were identified differently, with some participants reporting doing an individual goal-setting session before the group started, including the use of COPM. Others reported having all children attending a group having one common goal related to ball skills and two individual goals worked in the group sessions, or an individual session of CO-OP working in their individually identified goals followed by group sessions working on other people's goals as well. Whilst not all respondents used an outcome measure to evaluate outcomes of CO-OP (*n* = 12/160, 8%), the majority used the COPM (per the CO-OP protocol) to evaluate the intervention (*n* = 100/160, 63%). A lower proportion used the second recommended observational tool to evaluate CO-OP, the Performance Quality Rating Scale (PQRS) (20, 21) (*n* = 51/160, 32%) and a lower proportion used either the Goal Attainment Scale (GAS) (22) or informal goal setting (*n* = 24/160, 15%).

### 3.4 Challenges with using CO-OP in practice

Lack of time was perceived as one of the major barriers to implementing CO-OP with 75/106 (71%), followed by service constraints at 60% (64/106), and child factors at 59% (63/106). Family factors and confidence in applying the approach were also reported by 54 (51%) and 46 (43%) participants, respectively. Only 13 participants (12%) reported the effectiveness (or lack of) as a barrier to implementing the approach in routine practice.

When asked to elaborate on barriers, participants described a range of time constraints that impacted their ability to use CO-OP routinely in practice, including limitations in the number of sessions offered and difficulty implementing the full CO-OP approach within sessions, as well as referral pathway constraints impacting session timing and frequency (e.g., “*Time constraints within our service make it difficult to deliver the full CO-OP approach with fidelity*”, “*Limited session numbers and high caseloads mean we often have to use only parts of the approach*”). Participants also identified time constraints related to administrative and documentation demands. Others identified family-related time barriers, including families being unable to commit to regular sessions or difficulties scheduling suitable CO-OP group session times that aligned with children and young people's schedules. The teaching of CO-OP and CO-OP strategies were also raised as time barriers, together with constraints around limited sessions. CO-OP not being integrated into established service pathways was felt a barrier to implementing the approach with fidelity (e.g., “*CO-OP is not well integrated into our service pathways, which makes it challenging to use routinely*”)*.* Other service limitations to implementing the approach included a perceived incompatibility with delivering the approach in settings such as mainstream schools, psychiatric wards, or acute stroke units.

Participants also identified child and family-related barriers to implementing CO-OP. Examples of child-related barriers included difficulties identifying goals due to lack of motivation, difficulties articulating specific goals, or having goals imposed by parents and/or schools rather than child-driven. Other participants identified difficulties identifying appropriate clients in their caseloads: “*We do not always get the perfect client for CO-OP*”. Family-related barriers included the financial and time constraints implications for family having to attend multiple therapy sessions per week. Several participants identified that some parents were resistant to CO-OP's top-down, goal-oriented philosophy, preferring bottom-up impairment-focused approaches. Some participants also highlighted that sometimes families lacked an understanding of the commitment required to follow through with recommendations to enhance generalization and transfer to the home environment and how families seek immediate outcomes (e.g., “*Families sometimes struggle with the time commitment required to support generalisation at home*”).

Lack of confidence was particularly raised when working in services without other people trained on theapproach (e.g., “*It is harder to feel confident using CO-OP when you are the only trained therapist in the service*”) and there was a lack of support to understand if the approach was being delivered appropriately (e.g., “*I would benefit from feedback to know whether I am delivering the approach appropriately*”). Difficulties, particularly on the appropriate use of dynamic performance analysis and guided discovery were reported.

### 3.5 Supporting CO-OP practice

When exploring support and facilitators to use the approach, respondents reported how they maintained their skills in using CO-OP with (*n* = 16, 4.89%) participating in journal clubs, (*n* = 65, 19.89%) searching the literature, (*n* = 38, 11.62%) using research alerts, (*n* = 110, 33.64%) peer discussion, and (*n* = 52, 15.9%) accessed clinical supervision. Other examples included participating in a community of practice, teaching others, practice and reflection, utilizing available resources such as ICAN and referring to the course notes and (*n* = 23,12%) of respondents reported not doing any. Other aspects included “all” if to use it again, appropriate client base, case discussion of more complex cases, coaching with case studies, and refresher courses.

The majority of respondents (*n* = 112, 66.67%) would like further support to develop their CO-OP practice, with (*n* = 36, 11.9%) not sure and (*n* = 20, 11.9%) not needing further support. For those wanting further support who responded they suggested frequency of support monthly (*n* = 34, 30%), biannually (*n* = 35, 31%) and annually (*n* = 22, 20%). For the remainder of respondents, frequency was either unknown or suggested to be “ad hoc”. The specific areas of support related to applying the approach. Support in the form of a community of practice was reported most frequently as one method to support the continued development of their skills by (*n* = 91/133, 68%) (e.g., “*A community of practice would really help to share experiences and problem-solve challenges*”), followed by question-and-answer sessions (*n* = 80, 60%) and newsletter updates (*n* = 62, 47%). Paid refresher sessions, journal clubs, and study groups were also identified as useful support strategies by 38%–40% of the participants (e.g., “*Refresher sessions with case examples and video demonstrations would support continued skill development*”)*.* Other suggestions included expert support with direct observations of staff doing CO-OP getting direct feedback on performance, local supervision and modelling, case studies withmore video examples on the ICAN website, more adult resources, a quicker version, local community of practices, and coaching (e.g., “*More practical resources and simplified handouts to share with families would be useful*).”

## 4 Discussion

This international survey provides an overview of how CO-OP is currently implemented following formal training, highlighting patters of use, therapists perceptions, and key implementation challenges. Overall, findings indicate that CO-OP Is used across multiple international contexts but remains most commonly applied with children and young people. While therapists generally perceived the training as helpful and relevant, confidence and fidelity in implementation varied, with challenges related to time and service constraints, client readiness for goal setting and outcome measurement. Difficulties maintaining core elements of CO-OP; particularly client centred goals and use of the recommended outcome measures were evident. The findings strongly suggest that training alone is insufficient to ensure sustained implementation.

### 4.1 International use of CO-OP and populations served

This study provides important insights into the current and future implementation of the CO-OP approach in clinical practice following formal training. The findings demonstrate that CO-OP is used internationally, with respondents from multiple regions including the United Kingdom, the Netherlands, Australia, North America, and Asia. This reflects the growing international reach of the approach and aligns with the expanding evidence based for CO-OP across the lifespan when movement/coordination difficulties impact occupational performance and participation (3, 4, 7, 9).

Despite this broad evidence base, the survey results indicate that CO-OP is most commonly applied with children and young people. This likely reflects the historical focus of CO-OP training on paediatric populations, as well as the longer-standing evidence base in this group. Since 2009, evidence supporting the use of CO-OP with adults has emerged (23) and training has since adopted a lifespan perspective; however uptake with adult populations remains comparatively lower. In addition, although CO-OP training is available to multiple health professionals, occupational therapists continue to represent the vast majority of respondents (94%) suggesting that further efforts may be required to promote the approach across different professional groups working across the life span to ensure equitable access for all populations who may benefit.

### 4.2 Therapists' perceptions of usefulness and confidence following training

Consistent with other studies investigating the effect of training on implementation fidelity (24), considerable variation was reported with barriers identified that impacted implementation fidelity. Implementation barriers included time and service constraints, confidence, and child/family factors. Whilst most respondents felt CO-OP training prepared them very well or extremely well for their practice, confidence in implementing the approach varied significantly. One reason may be that almost half (46%) had completed the training in the past 1–2 years and might have had limited opportunities to apply the approach. Lack of confidence without other trained staff in their teams and further support were also mentioned as key factors affecting confidence. Whilst peer discussion, clinical supervision, or literature searches were reported as activities to support skill development, two-thirds wanted further support to develop their expertise. Further support was suggested including the use of a community of practice, question and answer sessions, newsletters, refresher sessions, and direct observation feedback. Confidence in using the approach was associated with the availability of appropriate clients, which would enable the therapist to gain experience and peer support and or supervision.

### 4.3 Fidelity, goal setting, and outcome measurement

One of the central aims of the CO-OP training is to support implementation with fidelity, including adherence to core features such as client-chosen goal setting and the use of recommended outcome measures. In this study, goal setting was identified as a particular challenge, especially when clients had difficulty articulating functional, meaningful goals. This challenge was reported across age groups including children and adults following stroke.

Although the majority reported using the COPM, given this is a “must do”, a small proportion (8%) reported using no outcome measures or using measures that do not measure occupational performance, such as the Beery Buktenica Test of Visual Motor Integration (25). More than half of the respondents (63%) reported using the COPM to measure the effect of the intervention, and only 32% measured performance changes with the observational rating tool, PQRS. The finding is noteworthy, given that outcome measurement with COPM and PQRS is central to evaluating the approach. This is in line with a recent review of the literature on the use of CO-OP in studies with children and young people showing 92% of studies reporting the use of the COPM and 61% of studies reporting the use of the PQRS (9).

Difficulties with goal setting may be influenced by clinician experience, parent or referrer expectations, and assumptions about client readiness. Ryan et al. 2024 reached consensus in a Delphi study exploring child -led goal setting (26). Readiness for goal setting with a strong agreement reached that an indication of being ready for goal setting was when “a child has interest in an activity or occupation”. Identifying functional goals for individuals following stroke was considered difficult for some “*most often they say, I want to improve my arm*” despite the solid evidence in this patient group including large implementation studies (12, 13, 27). Other believes of having “the ideal candidate” for CO-OP require further in-depth exploration as some participants reported not having appropriate clients such as autistic children despite the emerging evidence in this area (28), or the perceived incompatibility of delivering the approach in stroke units when the evidence indicates that those individuals with stroke receiving CO-OP as early intervention in stroke acute units have better results when compared to usual care (12). These findings suggest a need for enhanced training and resources focused on facilitating client-led goal identification across populations and contexts.

### 4.4 Barriers to implementing CO-OP in clinical practice

Time constraints were the most frequently reported barrier to implementing CO-OP (70% of respondents), consistent with previous literature, which has identified time constraints as a critical challenge to embedding these structured sessions into routine service delivery. Notably, the results also note as barriers the referral pathway restrictions, administrative demands, and the additional time required to teach CO-OP strategies. The later reason might indicate a misuse of how strategies are elucidated from clients and requires further exploration to ensure fidelity of delivering the approach. Some of these challenges may reflect misunderstandings about guided discovery and dynamic performance analysis, highlighting the need for ongoing support to maintain fidelity.

Service-level barriers, including a lack of integration of CO-OP into existing pathways and perceived incompatibility with certain settings (e.g., schools, acute stroke units), raises important questions around systemic and contextual barriers that may hinder the scalability of the approach. These findings underscored the importance to consider organizational and contextual factors with strong process evaluation and implementation-based research to scale and sustain CO-OP in diverse practice environments.

The contexts in which interventions take place are crucial to understanding how they work and if it can be generalized to other settings. Process evaluations focusing on the context for the implementation of the approach would be required in future studies. This would enable studies to consider important factors and design studies representative of the reality of clinical services delivering interventions. This might also allow for a better understanding of how to integrate CO-OP in current clinical pathways.

### 4.5 Supporting implementation and implications for practice

Consistent with implementation science literature, the findings reinforce that training alone is not sufficient to ensure successful implementation of CO-OP in practice (29, 30). Strategies that can support implementation of the approach may include mentorship programs, clinical supervision, coaching and community of practices (14, 30).

Reported CO-OP dosage was often lower than the recommended dosage in research and training consisting of 10 therapy sessions at least once a week, largely due to service and funding constraints. Group delivery models showed even greater variability, raising important questions about optimal dosage, efficiency, and outcomes across delivery formats. Future research is needed to explore minimum effective dosage and to compare individual and group-based CO-OP delivery in real-world settings.

### 4.6 Future research directions

Given the prominent role of child and family factors as barriers, further research is needed to understand the acceptability of CO-OP from the perspectives of children, young people, adults, and families. Co-designed research incorporating lived experience could inform adaptations to deliver formats, including blended or telehealth approaches, to enhance engagement and sustainability support adherence and acceptability of the approach. In addition, process evaluations examining contextual and organisational factors would support a deeper understanding of how CO-OP is implemented across settings and how it can be integrated into existing service pathways.

### 4.6 Limitations

A substantial proportion of respondents were based in the UK, with smaller but meaningful representation from other countries. Given international variation in health systems and clinical pathways, further research is needed to explore how contextual factors influence CO-OP implementation. Additionally, this study captured clinicians perspectives only; incorporating the views of service users and families will be essential for developing comprehensive implementation strategies. A small proportion of respondents (3%) reported using CO-OP without having completed what they identified as formal training. This likely reflects the historical absence of internationally governed certification processes and the use of informal or locally delivered training prior to the establishment of current governance structures.

## 5 Conclusions

This international survey demonstrates that CO-OP is widely used in clinical practice, particularly with children and young people, and is perceived by therapists as a useful and relevant approach following formal training. However, the findings highlight important challenges to implementation, including difficulties supporting clients to identify functional, meaningful goals, time and service constraints, and variability in confidence and fidelity when applying the full CO-OP approach. Although training was viewed positively, these results indicate that training alone is insufficient to ensure sustained and consistent implementation. Ongoing post-training support—such as supervision, communities of practice, and opportunities for feedback—appears necessary to address identified barriers and strengthen confidence, uptake, and fidelity. Future research should prioritize implementation-focused studies, including process evaluations that examine contextual factors, service pathways, and client and family perspectives, to optimize the delivery and impact of CO-OP in real-world clinical settings.

## Data Availability

The raw data supporting the conclusions of this article will be made available by the authors, without undue reservation.
