# Supplementary material for: International health professional perspectives of using the Cognitive Orientation to daily Occupational Performance approach in routine practice
Source: Front Rehabil Sci. 2026 Feb 26;7:1704831. doi: 10.3389/fresc.2026.1704831 (PMC12979492; doi:10.3389/fresc.2026.1704831)
Supplement: Supplementary file 1 [file Datasheet1.pdf]

English (United Kingdom)

Default Question Block

What is your professional background?

- [ ] Occupational Therapist
- [ ] Physiotherapist
- [ ] Speech and Language Therapist
- [ ] Exercise Physiologist
- [ ] Psychologist
- [ ] Psycho-motor therapist
- [ ] Other (please specify)

How long since you graduated in your profession?

0 3 5 8 11 13 16 19 21 24 27 29 32 35 37 40

0	3	5	8	11	13	16	19	21	24	27	29	32	35	37	40

Click to write Choice 1

What is the country where you work?

How many years since competing your CO-OP training?

- 1-2 years
- 3-5 years
- 6-10 years
- >10 years
- I have never had formal training

Who have you used CO-OP with?

- Children 5-18
- Young people 18-25

- [ ] Adults 26+
- [ ] Older people 65+

What diagnosis did children and young people have?

- [ ] Developmental Coordination Disorder
- [ ] Autism Spectrum Disorder
- [ ] Attention Deficit Hyperactivity Disorder
- [ ] Cerebral Palsy
- [ ] Spina Bifida
- [ ] Acquired Brain Injury
- [ ] Down Syndrome
- [ ] Any other intellectual disability
- [ ] Fetal Alcohol Spectrum Disorder
- [ ] Other (please specify)

What diagnosis did adults have?

- [ ] Stroke

- ☐ Acquired Brain Injury
- ☐ Cerebral Palsy
- ☐ Cognitive decline
- ☐ Parkinsons Disease
- ☐ Multiple Sclerosis
- ☐ Fatigue
- ☐ Mental health, please specify
- ☐ Long COVID
- ☐ Other (please specify)

What area of work are you currently employed in?

- ☐ Public health department – acute
- ☐ Public health department – community
- ☐ Public health department – schools
- ☐ Public health department – social care

☐ Public health department – mental health
☐ Independent/privately funded - acute setting
☐ Independent/privately funded - community setting
☐ Independent/privately funded - schools
☐ Independent - not for profit
☐ Other (please specify)

What age groups do you work with?

☐ <6 years
☐ 6-11 years
☐ 12-16 years
☐ 16-19 years
☐ 19-25 years
☐ 26-65 years
☐ >65 years

What CO-OP training have you completed?

- [ ] I haven't completed any CO-OP training
- [ ] I have been trained by colleagues
- [ ] 2 day course (face to face)
- [ ] 2 day course equivalent (virtual)
- [ ] ICAN online training
- [ ] Consolidation day (face to face)
- [ ] Consolidation day (virtual)
- [ ] Certification (face to face)
- [ ] Certification (vitual)
- [ ] Other (please specify)

Q10. Have you ever accessed the ICAN.org website?

- ○ yes
- ○ No
- ○ Not sure

What was the purpose of accessing ICAN.org website?

Was the website useful?
Any other information or suggestions for improving the website?

- ◯ Extremely useless
- ◯ Moderately useless
- ◯ Slightly useless
- ◯ Neither useful nor useless
- ◯ Slightly useful
- ◯ Moderately useful
- ◯ Extremely useful

Block 1

How well did the CO-OP training prepare you to understand the approach in your current practice

- ◯ Not well at all
- ◯ Slightly well
- ◯ Moderately well

- ○ Very well
- ○ Extremely well

Since completing the CO-OP training how confident do you feel in applying the CO-OP approach with your clients (0=not confident, 100= very confident)

	0	10	20	30	40	50	60	70	80	90	100
0=not confident, 100=very confident											

Are you currently using the CO-OP approach in your practice?

- ○ Yes
- ○ No

Could you tell us the reason why you are not using the CO-OP Approach?

Would you consider using it in the future?

How frequently do you use the full CO-OP approach?

- I always have a client on my caseload with whom I am using the CO-OP Approach
- I often have a client on my caseload with whom I am using the CO-OP Approach
- I occasionally have a client on my caseload with whom I am using the CO-OP approach
- I rarely have a client on my caseload with whom I am using the CO-OP approach
- I never have a client on my caseload with whom I am using the CO-OP approach

Do you deliver CO-OP individually and/or in groups?

- [ ] Individual
- [ ] Group

How many sessions do you offer when using the CO-OP approach individually?

If your service runs groups, how often do they run annually?

What is the maximum number of children per group? And what is the ratio of therapists and support staff to number of children?

What is the number of sessions if you run CO-OP in a group format?

How are goals set with the different participants?

What measures do you use to evaluate the intervention outcome?

Are there any particular barriers you face in putting CO-OP into practice? (tick all that apply)

- [ ] Confidence
- [ ] Time
- [ ] Service restraints
- [ ] Family factors
- [ ] Child factors
- [ ] Effectiveness
- [ ] Other (Please comment)

How are you currently maintaining and/or developing your skill using CO-OP (tick all that apply)

- ○ Journal clubs
- ○ Literature searches
- ○ Research alerts
- ○ Peer discussion
- ○ Clinical supervision

○ Other (please specify)

[ ]

Would you like further support to develop your CO-OP practice?

- ○ yes
- ○ no
- ○ not sure

What aspects of applying the approach would you like support with (tick all that apply)

- ☐ Theory - top down, systems theory, motor learning theory
- ☐ Key features - goal setting, DPA, Cognitive strategy use, guided discovery, involvement of significant others
- ☐ Case discussion
- ☐ Coaching on implementing CO-OP through observation
- ☐ Application of use with specific client groups
- ☐ Service issues - business cases, embed CO-OP with occupational performance approach, report and note writing

- [ ] Research updates/discussion
- [ ] Other (Please specify)

Block 2

What further support would enable you to progress your CO-OP practice? - tick all that apply

- [ ] Journal clubs
- [ ] Newsletter updates
- [ ] Study groups
- [ ] Question and answer sessions
- [ ] Community of practice
- [ ] Paid refresher sessions (half of full day)
- [ ] Other (please specify)

How often would you like to access extra learning and support?

Is there anything else you can think of what would be useful to further develop your use of the CO-OP approach in practice?

Powered by Qualtrics
